# Determination of some heavy metals in selected cosmetic products sold in kano metropolis, Nigeria

**DOI:** 10.1016/j.toxrep.2016.11.001

**Published:** 2016-11-22

**Authors:** Ali Sani, Maryam Bello Gaya, Fatima Aliyu Abubakar

**Affiliations:** Department of Biological Sciences, Bayero University Kano, Nigeria

**Keywords:** Heavy metals, Cosmetics, Concentration, Monitoring

## Abstract

The study was aimed at assessing the levels of some toxic metals in different cosmetic products sold at different shopping malls and markets in Kano Metropolis. The cosmetic items included ten face powder, ten skin lightening creams and ten lipsticks of various prices. The cosmetics were digested and analyzed for heavy metals (manganese, nickel, copper, cadmium, chromium and lead) using atomic absorption spectrophotometer. The range of the concentrations in skin lightening creams is 4.90–24.51, 3.68–11.03, 4.24–8.48, 0.14–1.32, 0–0.05 and 0.05–0.14 mg kg^−1^ for Mn, Ni, Cu, Cd, Cr and Pb respectively. The range in face powders are 4.90–44.12, 3.68–11.03, 4.24–8.48, 0.07–1.74, 0–0.03 and 0.08–0.33 mg kg^−1^ for Mn, Ni, Cu, Cd, Cr and Pb respectively. The concentration ranges in lipsticks are 2.45–22.06, 0–11.03, 4.24–12.71, 0.07–1.67, 0–0.05 and 0–0.19 mg kg^−1^ for Mn, Ni, Cu, Cd, Cr and Pb respectively. T test showed no statistical significant difference in concentrations of metals between the expensive and cheap cosmetic products. It is obvious from the present study that the use of these cosmetic products exposes users to low concentrations of toxic heavy metals which could constitute potential health risk to users since they are known to accumulate in biological systems over time. Similarly, regular monitoring of other heavy metals and chemicals used in the manufacture of cosmetics products which may cause health risks to users should be emphasized.

## 1 Introduction

A cosmetic is a substance or preparation used in contact with various parts of the human body such as epidermis, hair, nails, lips and external genital organs (external parts) or applied to the teeth or mucous membranes of the oral cavity with a view or for the purpose of cleaning, perfuming, protection, changing their outlook, converting body odours and keeping the surfaces in good condition [1], [2]. Cosmetics are mixtures of some ingredients such as surfactants, oils and they need to be effective, long lasting, stable and safe to human use [3].

The various forms of cosmetic include powder, rouge (used to colour the face, lighten and remove flaws to produce an impression of youth and health), lipstick and lip gloss (used to colour the lips), mascara (used to enhance the **eye** lashes), nail polish (used to colour the fingernails and toenails), eye liner and eye shadow (used to colour the eye lids) [2].

Very high level of trace metals were reported in locally produced facial makeup in Nigeria [4]. Similarly, herbal cosmetics preparations sold in Indian market had the concentrations of mercury in 2 of the samples and lead in 6 of the samples exceeding the WHO permissive limit for herbal cosmetics preparation [5].

In addition, talcum powder had varied concentration of lead (15–20 ppm) and chromium (20–30 ppm) [6], [7] also found that facial talcum powder contain asbestiform and substantial concentrations of nickel, cobalt and chromium.

Dermal exposure is considered to be the most significant route for cosmetic products since the majority of cosmetics are applied topically. Dermal absorption of heavy metals is fairly minimal. The absorption of individual metals is influenced by some factors which include: physical and chemical properties of the mixtures. Oral exposure can also occur especially for cosmetics used in and around the mouth as well as from hand-to-mouth contact after exposure to cosmetics containing these heavy metals. However, inhalation exposure is typically considered to be negligible [8].

Humans are exposed constantly, simultaneously and consecutively to large numbers of chemicals of diverse nature from various sources and via multiple routes [9]. Cosmetics, especially the skin lightening types are used widely in most African countries, especially by women. Since these products are used for a long time, on a large body surface and under hot humid conditions, cutaneous absorption is enhanced [10]. The complications of these products can be very serious. Some studies have indicated an association between some ingredients of cosmetics and a variety of health problems [11].

The pattern of accumulation of these metals in female body can be altered by some physiological changes. Most of the metals act as endocrine disrupters interfering with the hormonal system [12].

A study by [13] on the levels of lead, cadmium, nickel, chromium, and mercury was conducted have been assessed in 28 body creams and lotions, 10 powders, 3 soaps, 5 eye make-ups, and 4 lipsticks widely available on Nigerian markets. The increases over suggested or mandated levels of lead in these creams and lotions ranged from 6.1 to 45.9 and from 1.2 to 9.2 mg kg^−1^ when compared with Cosmetic Ingredients Review Expert Panel 2007 and German safe maximum permissible limit of lead in cosmetics, respectively. Chromium And mercury were undetected in 100% of the cosmetic product.

Faruruwa and Bartholomew [14] revealed that in a total of 40 samples consisting of 10 different types of facial cosmetics commonly used in Nigeria, Chromium, nickel, zinc and iron were found in varying concentrations in all the samples, 85% of the samples also contain Cd while 18 of the 40 samples have Pb above detection limit (0.20–31.70 mg kg^−1^). The levels of Pb and Cd in superstores and open markets were lower in comparison to other metals under considerations, while the concentration of Fe is generally high (72.90–261275.60 mg kg^−1^) with one of the powders having the highest value of 261275.60 mg kg^−1^. The lowest value 72.90 mg kg^−1^ was found in face cleanser.

In another study published in Kano State, Nigeria, a total of 20 skin powder products were sampled from Sabon Gari (Bata) market with the aim of detecting the presence of cadmium and chromium. All the metals were detected in all the samples, even though, at different concentrations (P ≤ 0.050). The concentration of chromium was lower when compared to cadmium [15].

Due to the spuriousness of these cosmetic products in developing and under developed countries such as Nigeria, the facial cosmetics are being sold under the brand name of well reputed national and international companies in both open markets and superstores in the country.

Considering the different conditions of sales, which could possibly affect the heavy metals content of these cosmetics, coupled with the lack of regulations on cosmetics in the country and also marred with counterfeiting of cosmetic products, both markets faces non- regulations and the unfounded believe that genuine and safer products are found in the bigger and more expensive market like the superstores than in the smaller and less expensive market like the open market. In addition, Kano is a well known state for its commercial activities spanning across various products including cosmetics. These cosmetics are been channeled to different parts of the country for use. However, the complete profile of heavy metals in cosmetics in the state is lacking. Similarly, there is a higher patronage of these products from people of different ages and level of literacy because of the immense quest for beauty. Therefore, the need arises to investigate the heavy metal contents of cosmetics in the state.

This research was aimed at determining the concentration of some heavy metals in cosmetic products in Kano metropolis through the determination of the concentration of Copper, Manganese, Nickel, Chromium, Cadmium and Lead in skin lightening creams, face powder and lipsticks. These heavy metals are among the most toxic heavy metals and their role in cosmetics preparation.

## 2 Materials and methods

### 2.1 Samples and sample collection

Ten samples each of skin lightening creams, face powder and lipsticks of different brands were bought from different cosmetics shops from local market and super markets in Kano. These categories of cosmetics are the most commonly used and from each category representative sample from various brands were selected. The samples were of different qualities and popular brands with different price ranges from expensive products and cheap products (that is higher and lower prices). They are presented in Table 1.

**Table 1:** Information on the types and brands of cosmetics used for the study.

Lightening creams			Face powders			Lipstick		
Brand name	Colour	Country	Brand name	Colour	Country	Brand name	Colour	Country
Pure skin	Pink	China	Ailin	Light brown	USA	Ms rose	Orange	Canada
Skin code body lotion	White	Cote d’Ivoire	Milani	Dark brown	Canada	Magic lipstick	Purple	France
Clair liss	White	Cote d’Ivoire	Mac	Dark brown	USA	Frank bee	–	–
Saga white tore	White	Nigeria	Iman	Dark brown	China	Jose face	Brown	France
Citrolight	Lemon	Cote d’Ivoire	Sleek	Light brown	USA	Island beauty	–	France
Peauclaire	White	Cote d’Ivoire	Maikay	Light brown	Mexico	Iman	–	China
Sivoclair	Yellow	Cote d’Ivoire	Fashion fair	Light brown	USA	Enchanteur	White	Nigeria
Bioclaire	White	Cote d’Ivoire	Black opal	Light brown	USA	Chun pair	White	China
Carotene	Yellow	Cote d’Ivoire	Lidanxiu	Light brown	Unknown	Casvyne collection	–	–
Labidjanaise	Yellow	Italy	Classic	Light brown	China	Chanteevi	Maroone	Unknown

### 2.2 Reagents and chemicals

Analytical grade nitric acid (65%, Sigma Aldrich) and perchloric acid (70%, Sigma Aldrich) were used for sample preparation. Standard solutions for calibration of Lead, Chromium, Cadmium, Copper, Manganese and Nickel were prepared from 1000 mg/l Standard Stock Solution of GFS Fishers’ AAS Reference Standard. All the solutions were prepared in distilled water. Dilution correction was applied for samples diluted or concentrated during analysis. These stock solutions were serially diluted to give concentrations as below:

**Cadmium**: A calibration curve with different cadmium concentrations (0, 0.1, 0.3 and 0.5 mg/l) was prepared.

**Chromium**: A calibration curve with different chromium concentrations (0, 0.5, 1.0 and 1.5 mg/l) was prepared.

**Lead**: A calibration curve with different lead concentrations (0, 1, 2 and 3 mg/l) was prepared.

**Manganese**: A calibration curve with different manganese concentrations (0, 1.5, 2.5 and 3.5 mg/l) was prepared.

**Nickel**: A calibration curve with different nickel concentrations (0, 0.2, 0.6 and 1.0 mg/l) was prepared.

**Copper**: A calibration curve with different copper concentrations (0.5, 1, 1.5 and 2 mg/l)

### 2.3 Sample digestion and chemical analysis

Glasswares and plastics were washed, rinsed many times with tap water and then soaked in 5% HNO_3_ solution for 24 h. They were then rinsed with deionized water. 3.0 g each of face powders and lipsticks were weighed into a porcelain crucible and dry-ashed in a muffle furnace by stepwise increase of the temperature up to 550 °C for 2 h. The ashed samples were digested with a 5 ml of IM HNO3 and then evaporated close to dryness on a hot plate in fuming hood. They were allowed to cool, filtered through whatmann no. 42 filter paper and were diluted up to the mark (100 ml) into a calibrated flask [16], [17]. Skin lightening creams were wet digested with a 4:1 mixture of nitric acid (65%) and perchloric acid (70%) on a hot plate in fuming hood near to dryness [16], [17] by slowly increasing the temperature for 3hr. The procedure was repeated through addition of mixture of acid by slow and continuous heating until the evolution of white fumes (marking the end of the digestion process) and near to dryness [18]. The solutions were allowed to cool, filtered by whatmann no. 42 into a calibrated flask (100 ml), and were diluted up to the mark. The sample solution was analyzed for Mn, Ni, Cu, Cd, Cr and Pb using Flame Atomic Absorption Spectrophotometer (210 VGP Atomic Absorption Spectrophotometer, BUCK SCIENTIFIC, East Norwalk, USA). The instruments working conditions and parameters were presented in Table 2.

**Table 2:** Working conditions for determination of concentration of some heavy metals using Atomic absorption spectrophotometer.

Metals	Wavelength (nm)	Slit width (nm)	Detection limit (mg/l)	Lamp current (mA)	Linear range (mg/l)	Flame type (colour)
Mn	279.5	0.7	0.03	10	2.50	A-A, lean/blue
Cd	228.80	0.7	0.01	2	2.00	A-A, lean/blue
Cr	357.90	0.7	0.04	2	5.00	A-A, rich/yellow
Cu	324.80	0.7	0.005	1.5	5.00	A-A, lean/blue
Pb	283.30	0.7	0.08	5	20.00	A-A, lean/blue
Ni	232.00	0.2	0.05	2.5	4.00	A-A, lean/blue

### 2.4 Data analysis

Student *t*-test was conducted to determine significant differences in concentration of metals between the two categories of each class of cosmetic. The software used was Sigmastat version 3.5, Systat software, California, USA Table 2.

## 3 Results and discussion

From Table 3, the mean ± SD concentration of Mn (17.65 ± 5.31) mg kg^−1^, Ni (8.09v3.08) mg kg^−1^, Cd (0.92 ± 0.023) mg kg^−1^ and Cr (0.016 ± 0.023) mg kg^−1^ in higher prices skin lightening creams have exceeded that of Mn (6.83 ± 1.98) mg kg^−1^, Ni (6.62 ± 4.03) mg kg^−1^, Cd (0.45 ± 0.43) mg kg^−1^ and Cr (0.00 ± 0.00) mg kg^−1^ respectively in lower price skin lightening creams. However, the mean ± SD concentration of Cu (5.94 ± 2.32) mg kg^−1^ in lower price is more than Cu (4.24 ± 0.00) mg kg^−1^ in higher price. In addition, the mean ± SD concentration of Pb (0.088 ± 0.038) mg kg^−1^ in higher price skin lightening creams is the same as in lower price creams.

**Table 3:** Concentrations (in mg kg^−1^) of some heavy metals in skin lightening creams in Kano.

Metals	Higher price			Lower price		
	Mean ± SD	Median	Range	Mean ± SD	Median	Range
Mn	17.65 ± 5.31	14.71	12.25	6.83 ± 1.98	7.35	4.71
Ni	8.09 ± 3.08	7.35	7.35	6.62 ± 4.03	3.68	7.35
Cu	4.24 ± 0.00	4.24	0	5.94 ± 2.32	4.24	4.24
Cd	0.92 ± 0.376	0.76	0.76	0.45 ± 0.43	0.42	1.11
Cr	0.016 ± 0.023	0	0.05	0.00 ± 0.00	0	0
Pb	0.088 ± 0.038	0.1	0.09	0.088 ± 0.038	0.1	0.09

In skin lightening creams, Mn is present in high concentration with a range between 4.90 to 24.51 mg kg^−1^ in skin lightening creams and that of Ni range from 3.676 to 11.029 mg kg^−1^. However [16], revealed that the range for the concentration of Ni in medicated and non medicated creams is 0 μg/g. The concentration of Cu range from 4.237 to 8.475 mg kg^−1^. The concentration of Cd range from 0 to 1.32 mg kg^−1^ and was not detected in 10% of the samples. The concentration of Cr range from 0 to 0.05 mg kg^−1^ and was not detected in 80% of the samples Similarly [19], did not detect Cr in all samples of skin cream. The concentration of Pb ranges from 0.05 to 0.14 mg kg^−1^ [16] found that the range for the concentration of Pb in medicated and non-medicated creams is 0 μg/g.

In Table 4, the mean ± SD concentration of Mn (27.45 ± 14.23) mg kg^−1^ and Cr (0.012 ± 0.016) mg kg^−1^ in higher price face powders have exceeded Mn (21.08 ± 11.05) mg kg^−1^ and Cr (0.00 ± 0.00) mg kg^−1^ respectively in lower price face powders. However, the mean ± SD concentration of Ni (8.82 ± 4.19) mg kg^−1^, Cu (6.78 ± 2.32) mg kg^−1^, Cd (0.96 ± 0.57) mg kg^−1^ and Pb (0.17 ± 0.47) mg kg^−1^ in lower price face powders have exceeded Ni (8.09 ± 4.79) mg kg^−1^, Cu (4.24 ± 0.00) mg kg^−1^, Cd (0.42 ± 0.47) mg kg^−1^ and Pb (0.13 ± 0.042) mg kg^−1^ respectively in higher price face powders.

**Table 4:** Concentrations (in mg kg^−1^) of some heavy metals in face powder in Kano.

Metals	Higher price			Lower price		
	Mean ± SD	Median	Range	Mean ± SD	Median	Range
Mn	27.45 ± 14.23	29.41	39.22	21.08 ± 11.05	19.61	26.97
Ni	8.09 ± 4.79	11.03	11.03	8.82 ± 4.19	7.35	11.03
Cu	4.24 ± 0.00	4.24	0	6.78 ± 2.32	8.48	4.24
Cd	0.42 ± 0.47	0.35	1.18	0.96 ± 0.57	1.04	1.6
Cr	0.012 ± 0.016	1	0.03	0.00 ± 0.00	0	0
Pb	0.13 ± 0.042	0.14	0.11	0.17 ± 0.12	0.19	0.33

Analysis of face powders showed that concentration of Mn ranges from 4.90 to 44.12 mg kg^−1^. The concentration of Ni ranges from 7.35 to 14.71 mg kg^−1^ and that of Cu ranges from 4.24 to 8.48 mg kg^−1^. In a study by [7], the range of concentration of Ni and Cu was 0 μg/g. The concentration of Cd ranges from 0 to 1.74 mg kg^−1^ and it was not detected in 10% of samples. Cr was not detected in 80% of samples though the concentration ranges from 0 to 0.03 mg kg^−1^. In a similar study by [7], the concentration of Cr ranges from 0 to 2.7 μg/g and that of Cd ranges from 0 to 8.1 μg/g. However, the concentration of Pb ranges from 0.08 to 0.33 mg kg^−1^ [7] found the range for concentration of Pb to be from 0.4 to 41 μg/g.

In Table 5, the mean ± SD concentration of Mn (13.73 ± 2.20) mg kg^−1^ and Ni (8.24 ± 3.29) mg kg^−1^ in lower price lipstick have exceeded the mean ± SD of Mn (11.27 ± 9.27) mg kg^−1^ and Ni (5.14 ± 4.19) mg kg^−1^ respectively in higher price lipstick. However, the mean ± SD of Cd (0.89 ± 0.58) mg kg^−1^, Cr (0.016 ± 0.023) mg kg^−1^ and Pb (0.106 ± 0.084) mg kg^−1^ in lower price lipstick have exceeded the mean ± SD concentration of Cd (0.34 ± 0.20) mg kg^−1^, Cr (0.00 ± 0.00) mg kg^−1^ and Pb (0.06 ± 0.042) mg kg^−1^ respectively in higher price lipstick. In addition, the mean ± SD concentration of Cu (7.63 ± 4.64) mg kg^−1^ in higher price lipstick is nearly the same as in lower price with mean ± SD concentration of Cu at (7.63 ± 3.54) mg kg^−1^.

**Table 5:** Concentration (in mg kg^−1^) of some heavy metals in lipstick in Kano.

Metals	Higher price			Lower price		
	Mean ± SD	Median	Range	Mean ± SD	Median	Range
Mn	13.73 ± 2.20	14.71	4.91	11.27 ± 9.27	9.8	19.61
Ni	8.24 ± 3.29	11.03	7.35	5.15 ± 4.19	3.68	11.03
Cu	7.63 ± 4.64	4.24	8.47	7.63 ± 3.54	8.48	8.47
Cd	0.34 ± 0.20	0.35	0.56	0.89 ± 0.58	0.83	1.39
Cr	0.00 ± 0.00	0	0	0.016 ± 0.023	0.1	0.05
Pb	0.06 ± 0.042	0.05	0.1	0.106 ± 0.084	0.1	0.19

Analysis of lipstick showed that the concentration of Mn ranges from 2.45 to 22.06 mg kg^−1^ while the concentration of Ni ranges from 3.68 to 11.03 mg kg^−1^. In a related study by [20], he reported a concentration range for Ni to be 7 to 22.8 μg/g. The concentration of Cu ranges from 4.24 to 12.71 mg kg^−1^. However, in a study by [20] the range for the concentration of Cu was found to be 0 μg/g. In addition, the concentration of Cd ranges from 0.07 to 1.67 mg kg^−1^. [20], reported that the average of cadmium levels in several facial cosmetics (eye cosmetics, lipsticks, and lip gloss) was approximately 1 μg/g. [21] also showed that cadmium content in all brands and colors of lipsticks was within the range of 0.200–0.500 μg/g. The amount of cadmium in the upper limit of the present study when compared with that of the above study was higher. Cr was not detected in 80% of the samples, though the concentration ranges from 0 to 0.05 mg kg^−1^. However, [20] found the range for the concentration of Cr to be 20.5 to 58.8 μg/g. The concentration of Pb ranges from 0.05 to 0.19 mg kg^−1^. Studies conducted by [20], [21], [22] showed that concentrations of lead in all brands of lipsticks analyzed were up to 41.1 μg/g 87–123 μg/g and 0.286–6.234 μg/g respectively. However, in the present study, Pb was not detected in 20% of the samples and has not exceeded the WHO permissible limit in 80% of the samples. Similarly [23], have found concentration of Pb in lipstick lower than what was observed by [20], [21], [22]. Cr was not detected in 80% of all the cosmetic products analyzed. However [24], were able to detect Cr in 50% of the cosmetic products analyzed in the range of 0.45 mg kg^−1^ to 17.83 mg kg^−1^. Pb and Cd were not detected in all the cosmetic products analyzed [24].

The concentration of the heavy metals analyzed are in the order Mn > Ni > Cu > Cd > Pb > Cr. Mn has the highest concentration while Cr has the least. [25] also found that Cr has the least concentration among all the heavy metals analyzed in cosmetic products.

*T* test showed no significant difference statistically between the higher and lower price cosmetics in terms of the concentration of the heavy metals analyzed (p < 0.05).

## 4 Conclusion and recommendations

From the results obtained, all the metals analyzed were detected. However, Cr was not detected in most of the samples. Mn has the highest concentration and Cr has the least. No statistical difference was observed between the higher and lower price cosmetic products in terms of the metals analyzed. It is however feared that the excessive use of cosmetic products contaminated with toxic heavy metals may lead to slow release of the metals into the human body and accumulate in body tissues and hence cause certain health complications. The following actions are recommended:

1 Regular monitoring of other heavy metals and chemicals used in the manufacture of cosmetics products which may cause health risks to users should be emphasized.
2 Regulatory guidelines on heavy metals in cosmetics should be formulated and enforced by relevant authorities in the manufacture of cosmetic products in Nigeria.
3 Public enlightment should be organized on the harmful effects of excessive or extensive use cosmetic products.
4 Laws should be enacted in order to limit the content of heavy metals in cosmetics and other household products and items.

## References

[1] Oyedeji F.O., Hassan G.O., Adeleke B.B. (2011). Hydroquinone and heavy metals levels in cosmetics marketed in Nigeria. Trends Appl. Sci. Res..

[2] Reed S.I. (2004). Cosmetics and Your Health. http://www.womens%20health%20govl/faq/cosmetics-%20your%20health.pdf.

[3] Roa N., Pathriba S. (1998). Cosmetics and Personal Care Products.

[4] Ajayi S.O., Oladipo M.O.A., Ogunsuyil H.O., Adebayo A.O. (2002). Determination of minor & trace elements in Birniwa tin pyrite and ornamental lead/zinc ore using neutron activation analysis. Bull. Chem. Soc. Ethiopia.

[5] Kumar S., Singh J., Garg M. (2012). AAS Estimation of heavy metals and trace elements in Indian herbal cosmetics preparations. Res. J. Chem. Sci..

[6] Gondal M.A., Seddigi Z.S., Nasir M.M., Gondal B. (2010). Spectroscopic detection of health hazardous contaminant in lipstick using Laser induce breakdown Spectroscopy. J. Hazard. Mater..

[7] Nnorom I.C. (2011). Trace metals in cosmetic facial talcum powders marketed in Nigeria. Toxicol. Environ. Chem..

[8] Sainio E., Jolanki R., Hakala E., Kanerva L. (2000). Metals and arsenic in eye shadows. Contact Dermat..

[9] Krois J.M., Mats R., Angela S., Dirk W. (2007). Embodiment in Cognition and Culture.

[10] Slodownik D., Lee A., Nixon R. (2008). Irritant contact dermatitis: a review. Australas. J. Dermatol..

[11] Nohynek G.J., Antignac E., Re T., Toutain H. (2010). Safety assessment of personal care products/cosmetics and their ingredients. Toxicol. Appl. Pharmacol..

[12] Bergamaschi L., Izzio E.R., Giaver I.G., Loppi M.S., Gallo R. (2007). Comparision between the accumulation capacity of four lichen species transplanted to an urban site. Environ. Pollut..

[13] Orisakwe O.E., Otaraku J.O. (2012). Metal concentrations in cosmetics commonly used in Nigeria. Sci. World J. Chem. Technol..

[14] Faruruwa M.D., Bartholomew S.P. (2014). Study of heavy metals content in facial cosmetics obtained from open markets and superstores within Kaduna metropolis, Nigeria. Am. J. Chem. Appl..

[15] Imam T.S., Sa’idu B.M. (2015). Detection of some toxic metals in selected brands of skin powders sold in kano metropolis market, northern Nigeria. IOSSR J. Environ. Sci. Toxicol. Food Technol. (IOSR- JESTFT).

[16] Ayenimo J.G., Yusuf A.M., Adekule A.S., Makinde O.W. (2010). Heavy metal exposure from personal care product. Bull. Environ. Contam. Toxicol..

[17] Saeed M., Muhammad N., Khan H. (2011). Assessment of heavy metal content of branded Pakistani herbal products. Trop. J. Pharm. Res..

[18] Theresa O.C., Onebunne O.C., Dorcas W.A., Ajani O.I. (2011). Potentially toxic metals exposure from body creams sold in Lagos Nigeria. Researcher.

[19] Ullah H. (2013). Comparative study of heavy metals content in cosmetic products of different countries marketed in Khyber Pakhtunkhwa, Pakistan. Arab. J. Chem..

[20] Nnorom C., Igwe J.C., Oji-Nnorom C.G. (2005). Trace metal contents of facial (make up) cosmetics commonly used in Nigeria. Afr. J. Biotechnol..

[21] Khalid A., Bukhari I.H., Riaz M. (2013). Determination of Lead, Cadmium, Chromium, and Nickel in different brands of lipsticks. Int. J. Biol. Pharm. Allied Sci..

[22] Tsankov I., Iordanova I., Lolova D., Uzunova S., Dinoeva S. (1982). Hygienic evaluation of the content of heavy metals (lead and copper) in cosmetic products. Probl. Khig..

[23] Nourmoradi H., Foroghi M., Farhadkhani M., Vahid D.M. (2013). Assessment of lead and cadmium levels in frequently used cosmetic products in Iran. J. Environ. Public Health.

[24] Ramakant S., Poornima S., Sapna J. (2014). Heavy metals in cosmetics. Centre for Science and Environment.

[25] Umar M.A., Caleb H. (2013). Analysis of metals in some cosmetic products in FCT abuja, Nigeria. Int. J. Res. Cosmet. Sci..

